# The Roles of Autophagy in Acute Lung Injury Induced by Myocardial Ischemia Reperfusion in Diabetic Rats

**DOI:** 10.1155/2018/5047526

**Published:** 2018-04-03

**Authors:** Liying Zhan, Yuan Zhang, Wating Su, Qiongxia Zhang, Rong Chen, Bo Zhao, Wei Li, Rui Xue, Zhongyuan Xia, Shaoqing Lei

**Affiliations:** Department of Anaesthesiology, Renmin Hospital of Wuhan University, Wuhan, China

## Abstract

Patients with diabetes are vulnerable to myocardial ischemia reperfusion (IR) injury, which may also induce acute lung injury (ALI) due to overaccumulation of reactive oxygen species (ROS) and inflammation cytokine in circulation. Despite autophagy plays a significant role in diabetes and pulmonary IR injury, the role of autophagy in ALI secondary to myocardial IR in diabetes remains largely elusive. We aimed to investigate pulmonary autophagy status and its roles in oxidative stress and inflammation reaction in lung tissues from diabetic rats subjected to myocardial IR. Control or diabetic rats were either treated with or without autophagy inducer rapamycin (Rap) or autophagy inhibitor 3-methyladenine (3-MA) before myocardial IR, which was achieved by occluding the left anterior descending coronary artery for 30 min and followed by reperfusion for 120 min. Diabetic rats subjected to myocardial IR showed more serious ALI with higher lung injury score and WET/DRY ratio and lower PaO_2_ as compared with control rats, accompanied with impaired autophagy indicated by reduced LC-3II/LC-3I ratio and Beclin-1 expression, decreased superoxide dismutase (SOD) activity, and increased 15-F2t-Isoprostane formation in lung tissues, as well as increased levels of leukocyte count and proinflammatory cytokines in BAL fluid. Improving autophagy with Rap significantly attenuated all these changes, but the autophagy inhibitor 3-MA exhibited adverse or opposite effects as Rap. In conclusion, diabetic lungs are more vulnerable to myocardial IR, which are involved in impaired autophagy. Improving autophagy could attenuate ALI induced by myocardial IR in diabetic rats, possibly through inhibiting inflammatory reaction and oxidative stress.

## 1. Introduction

Patients with diabetes often die from diabetes-related complications. Impaired control of blood glucose may lead to pathological changes and functional damages in numerous tissues and organs, including the eye, kidney, cardiovascular system, and nerve tissue [1]. Additionally, chronic abnormalities of glycaemia could also induce pulmonary dysfunction, which has aroused wide concern about the proposal of “diabetic lung” [2]. The pathogenesis of diabetic lung is implicated with hyperglycemia-induced oxidative stress and inflammatory reaction, which accelerate decline in respiratory function [3]. Although the practical implications of diabetic lung are subclinical, patients with diabetes are vulnerable to severe respiratory derangements when they underwent an acute or chronic pulmonary and/or cardiac disease [2, 3]. Acute myocardial infarction, a serious type of cardiac diseases, is one of the leading causes of diabetic mortality [4]. Effective and timely reperfusion therapies are essential for myocardium to survive from acute ischemia, but reperfusion after ischemia may induce myocardial ischemia reperfusion (IR) injury. Previous studies indicated that myocardial IR could also cause injury in distant organs, and the lung can be one of the most affected organs [5, 6]. Further studies suggested that acute lung injury (ALI) induced by myocardial IR in diabetes was more serious than that in nondiabetics [7]. However, there are little relevant reports concerning the potential mechanism of ALI induced by myocardial IR under diabetic condition.

Numerous studies employing both dietary and genetic animal models of diabetes have demonstrated that autophagy dysfunction were strongly associated with diabetic complications [8]. Autophagy is a conserved cellular catabolic process to preserve cellular homeostasis and survival. The key processes of autophagy, such as autophagosome biogenesis, lysosomal fusion, and cargo degradation, are collectively referred to as autophagic flux. Accumulating evidences have demonstrated that autophagy participates in the pathological process of ALI induced by pulmonary IR. Autophagy flux was elevated during the ischemia period and was enhanced significantly during reperfusion in the lung tissue of nondiabetic subject [9], and inhibition of this excessive autophagy status might alleviate lung injury induced by pulmonary IR [9, 10]. Also, there are some contrary conclusions that pulmonary IR impaired lung autophagy status and moderately promoting the impaired autophagy status is also beneficial to reduce lung IR injury [11]. All these findings suggest maintaining a moderate level of autophagy is the key to reduce lung injury. However, little is known about the role of autophagy in ALI secondary to myocardial IR in diabetic condition. Therefore, our present study mainly focused on the autophagic changes in lung tissues in diabetic rats subjected to myocardial IR. As the importance of oxidative and inflammatory stress in lung injury in diabetes [3], we also investigated the role of autophagy in oxidative stress and inflammation reaction in diabetic lung.

## 2. Materials and Methods

### 2.1. Experimental Animals

Male 8-week-old Sprague–Dawley rats were obtained from the Laboratory Animal Services Centre of Wuhan University. Rats were housed in the Centralized Animal Facilities of Wuhan University and allowed to access standard diet and water. All experimental procedures and protocols in this study were performed in accordance with the institutional animal care guidelines and approved by the Committee for Use of Live Animals in Teaching and Research. Food consumption and blood glucose levels were monitored weekly and water intake was assessed daily.

### 2.2. Induction of Diabetes

Diabetic model was induced via a single intraperitoneal injection with streptozotocin (STZ, Sigma, USA) at a dose of 60 mg/kg body weight, whereas normal control rats were injected with the same volume of mother solution (pH 4.5, Citrate buffer). Three days after the STZ administration, fasting blood glucose consistently exceeded 16.7 mmol/L was confirmed as diabetic status. All invasive operations in our experiment were performed under anesthesia with 3% pentobarbital sodium (50 mg/kg body weight) (Sigma-Aldrich, USA).

### 2.3. Experimental Protocol

Eight weeks after diabetic induction, myocardial IR operation was established as previously [12]. Briefly, the rats were anaesthetized and subjected to myocardial IR achieved by occluding the left anterior descending coronary artery (LAD) for 30 min and followed by reperfusion for 120 min. Sham-operated rats underwent the same surgical procedures without ligation. Diabetic rats were either pretreated with 3-methyladenine (3-MA, 15 mg/kg, intraperitoneally) [13] 30 min before ischemia or intravenous rapamycin (Rap, 0.25 mg/kg) [14] (Selleck Chemicals, USA) 15 min before ischemia or equal volume of saline for control. At the end of reperfusion, myocardial infarct size was measured using TTC (1% 2,3,5-triphenyltetrazoliumchloride) staining as previously described [12]. Myocardial infarct size was expressed as a percentage of the area at risk (AAR).

### 2.4. Blood Gas Analysis, WET/DRY Ratio, and HE Staining

We adopt the carotid blood gas analysis (partial pressure of oxygen, PO2) at 2 h of reperfusion, the wet/dry ratio, and the hematoxylin and eosin (HE) staining to assess pulmonary injury. Lung tissue was weighed and dried in an oven at a constant temperature of 60°C for 48 h to obtain a dehydrate consistency, then weighed again. Twice weight ratio was calculated as an indicator of edema. The specimens of the left lower lobe of the lung tissue were fixed, sectioned, and stained with HE and microscopic examination as described previously and quantify damage by lung injury score. The criteria of lung injury scores were based on five indexes: pulmonary interstitial edema, airway epithelial injury, hyaline membrane formation, neutrophil infiltration, and alveolar hemorrhage. Those indexes were graded as follows: normal = 0′, minimal change = 1′, mild change = 2′, moderate change = 3′, and severe change = 4′. Each section had five scores, and the scores for each criterion was recorded to evaluate lung tissue injury degree [15].

### 2.5. Leukocyte Count and Tumor Necrosis Factor- (TNF-) *α*, Interleukin- (IL-) 6, and IL-8 Detection in Broncho Alveolar Lavage (BAL) Fluid

BAL fluid was collected by cannulating the trachea with repeated 200 uL of sterile phosphate buffer saline (PBS) containing heparin up to a total volume of 1.0 mL. The BAL fluid was centrifuged at 4000*g* for 5 min at 4°C to separate the cells in the BALF from the liquid. The precipitated cells were resuspended in PBS and counted using a hemocytometer, and the leukocytes were counted with optical microscopy. The levels of TNF-*α*, IL-6, and IL-8 in BAL fluid were determined by using the corresponding kits (Jiancheng Bioengineering Institute, Nanjing, China). All experiments were performed according to the manufacturer's instructions.

### 2.6. Determination of Creatine Kinase Isoenzyme (CK-MB), 15-F2t-Isoprostane, and Superoxide Dismutase (SOD) Activity

20 mg of sample tissue were minced and homogenized in ice-cold RIPA (radioimmunoprecipitaion assay) buffer (Sigma, C0278). Homogenates were subject to centrifugation at 13000*g* for 15 min at 4°C to obtain the supernatant as sample tissue total protein preparation. The protein concentration was measured with a BCA (bicinchoninic acid) protein assay kit (Beyotime Biotechnology, Shanghai, China). Lung tissue SOD, 15-F2t-Isoprostane (15-F2t-IsoP), and plasma CK-MB (at 2 h of reperfusion) were measured using the corresponding kits (Jiancheng Bioengineering Institute, Nanjing, China). All experiments were performed according to the manufacturer's instructions.

### 2.7. Immunoblotting

Equal amounts of protein from each group were separated by 7.5–15% SDS-PAGE (sodium dodecyl sulfate polyacrylamide gel electrophoresis) and transferred to PVDF (polyvinylidene fluoride) membranes. Subsequently, the membranes were blocked and incubated overnight in a shaker with primary antibodies (LC-3, Beclin-1, and GAPDH, Cell Signaling Technology, 1 : 1000), respectively. After incubating with appropriate secondary antibodies (Cell Signaling Technology, 1 : 10,000), the membranes were subject to analyze by the Odyssey system (Germany). Data are presented as percent change relative to the measurement in control rats.

### 2.8. Statistical Analysis

Results were expressed as mean ± SD. GraphPad Prism software program (GraphPad Software Inc., San Diego, CA, USA) was used for statistical analysis. The statistical significance of differences (*P* < 0.05) was assessed by two-way repeated measures or one-way ANOVA analyses followed by Tukey's posthoc test wherever applicable.

## 3. Results

### 3.1. Myocardial Injury Secondary to Myocardial IR in Control and Diabetic Rats

**Figure 1:**
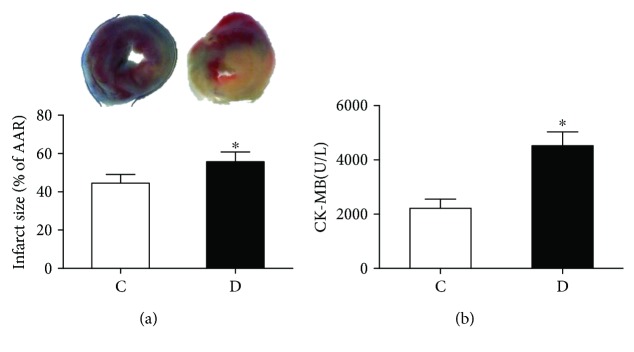
Control (C) or streptozotocin-induced diabetic (D) rats were subjected to 30 min of myocardial ischemia followed by 2 h of reperfusion. (a) Myocardial infarct size. (b) Plasma CK-MB levels. All values are expressed as means ± S.D., *n* = 8. ^∗^*P* < 0.05 versus C group.

At the end of this study, the STZ-induced diabetic rats showed significantly increased water intake, food consumption, and plasma glucose and loss of body weight as compared with control rats (Table 1). After the rats subjected to myocardial IR, as shown in Figure 1, the infarct size in diabetic rats was higher than that in control rats (*P* < 0.05), as well as the levels of CK-MB, indicating the diabetic rats are vulnerable to myocardial IR.

### 3.2. Diabetes Aggravates ALI Secondary to Myocardial IR, Accompanied with Impaired Autophagy Status

**Figure 2:**
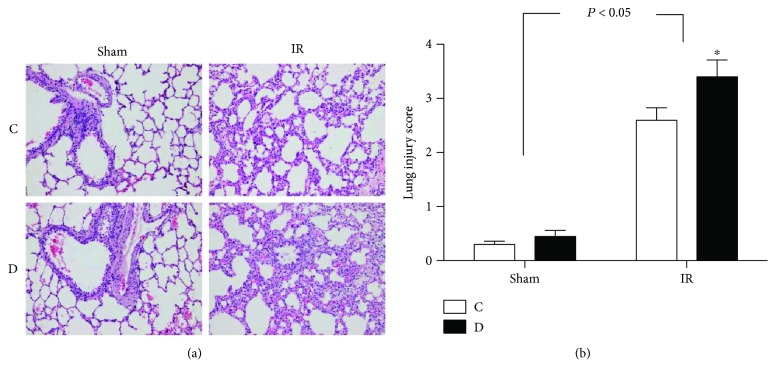
Histopathologic changes in rat lungs from control and diabetic rats subjected to sham or myocardial ischemia reperfusion (IR) operation under light microscopy. (a) Representative photomicrographs of hematoxylin and eosin (HE) staining (original magnification ×200). (b) Severity of lung injury expressed as injury score. All values are expressed as means ± S.D., *n* = 8. ^∗^*P* < 0.05 versus the corresponding C group.

The severity of lung injury was evaluated by standard HE staining and graded as injury score. There was no apparent pathologic alteration in the lung sections from the sham group in control or diabetic rats; however, after the rats subjected to myocardial IR insult, ALI with intra-alveolar and interstitial edema, hemorrhage, and inflammatory cell infiltration was showed in the lung section. These factors were more serious in diabetic rats. Hence, diabetic rats obtained a higher lung injury score than control rats (Figure 2).

**Figure 3:**
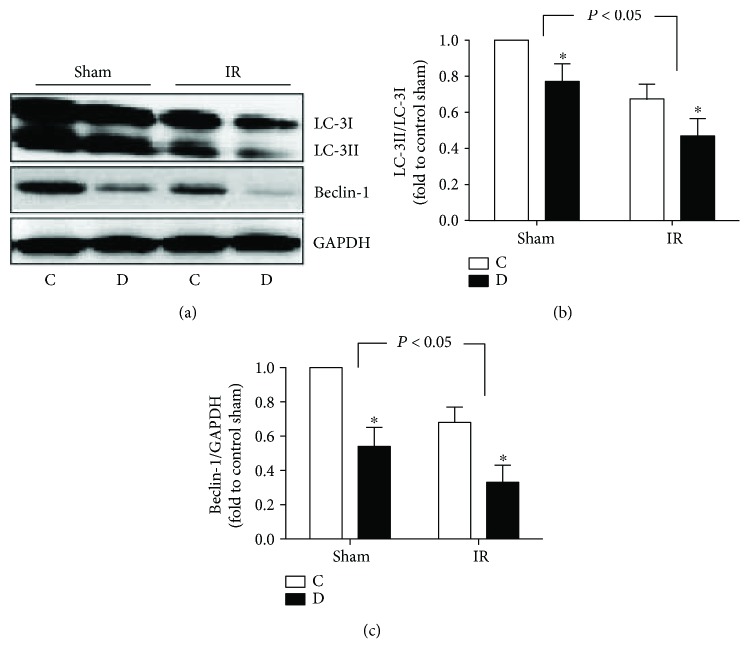
Expression of LC-3II and Beclin-1 in pulmonary tissues form control (C) and diabetic (D) rats subjected to sham or myocardial ischemia reperfusion (IR) operation. (a) Representative Western blot demonstrating LC-3II, LC-3I, and Beclin-1 expression with GAPDH as loading control. (b) LCII/LCI ratio were calculated by relative densitometric values and expressed as fold to control sham group. (c) Beclin-1 express were calculated by relative densitometric values and expressed as fold to control sham group. All values are expressed as mean means ± S.D., *n* = 8. ^∗^*P* < 0.05 versus the corresponding C group.

As the important role of autophagy in preserving cellular homeostasis and survival, we next measured autophagy status in lung tissues from control and diabetic rats subjected to myocardial IR. Autophagy begins with the activation of the Beclin-1 and sequences with the conjugation of membrane-bound LC-3II to autophagyosome. Thus, the Beclin-1 and LC-3II have been the indicators of autophagic initiation. As shown in Figure 3, the ratio LC-3II/LC-3I and Beclin-1 expression were significantly lower in diabetic rats than that in control rats, which were further decreased by myocardial IR (Figure 3), indicating both diabetic condition and myocardial IR insult impair autophagy status.

### 3.3. Effects of Rap and 3-MA on ALI Secondary to Myocardial IR in Diabetic Rats

**Figure 4:**
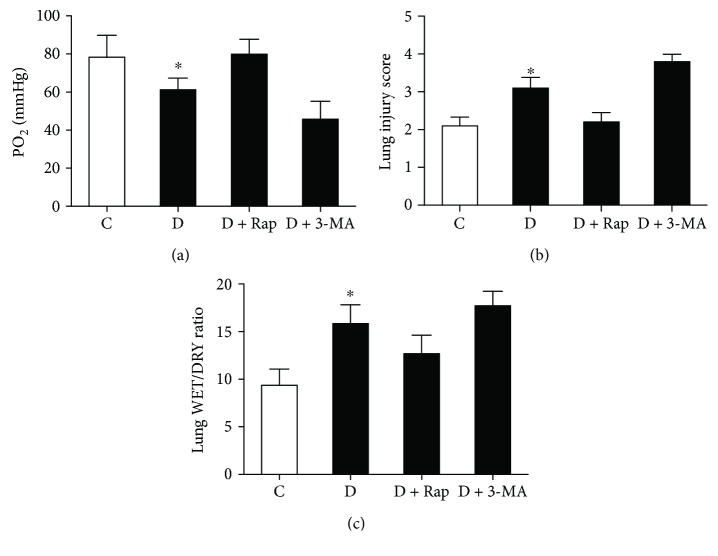
Effects of rapamycin (Rap) and 3-methyladenine (3-MA) on carotid blood partial pressure of oxygen (PO_2_) (a), Lung injury score (b), and WET/DRY ratio (c) in control and diabetic rats undergoing myocardial ischemia reperfusion (IR). All values are expressed as means ± S.D., *n* = 8. ^∗^*P* < 0.05 versus the other groups.

According to the above findings, it is still unknown the specific roles of autophagy in ALI in diabetic rats secondary to myocardial IR. Therefore, we next used autophagy inducer Rap and its inhibitor 3-MA to treat the diabetic rats. In the event of concern regarding the pulmonary function of rats during myocardial IR, the carotid blood gases were then measured. As shown in Figure 4(a), diabetic rats subjected to myocardial IR existed a significantly lower carotid blood PO_2_ as compared with control rats. Diabetic rats obtained a higher lung injury score than nondiabetic rats (Figure 4(b)). Similar trends were observed the lung tissue WET/DRY ratio (Figure 4(c)). Remarkably, all these changes were significantly attenuated by the autophagy inducer Rap but were further increased by autophagy inhibitor 3-MA.

### 3.4. Effects of Rap and 3-MA on Pulmonary Inflammation and Oxidative Stress in Diabetic Rats Subjected to Myocardial IR

**Figure 5:**
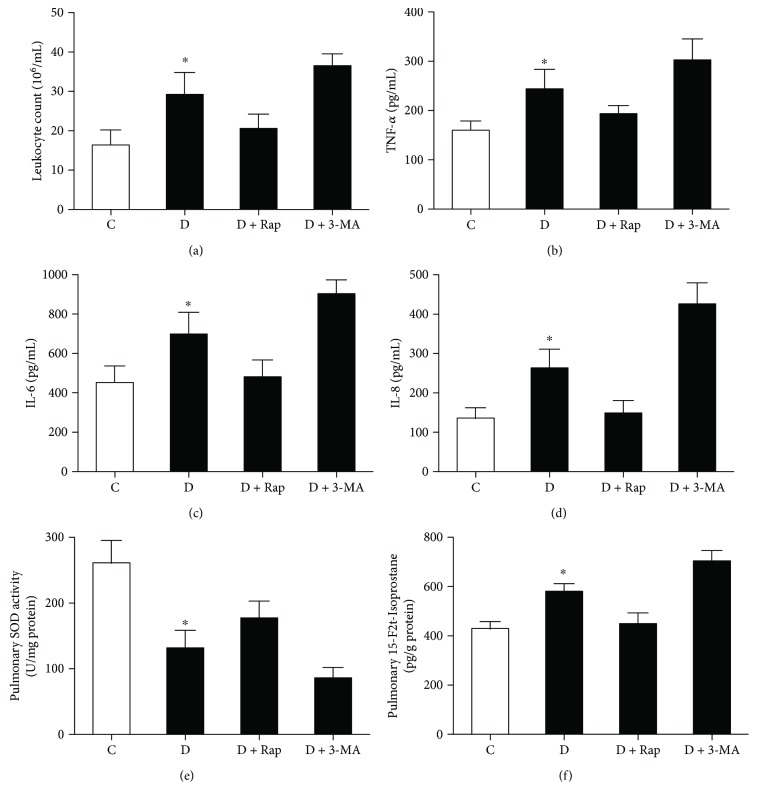
Effects of rapamycin (Rap) and 3-methyladenine (3-MA) on leukocyte count (a), TNF-*α* (b), IL-6 (c); IL-8 (d) in BAL fluid; and SOD (e) and 15-F2t-Isop (f) in lung tissues from control and diabetic rats undergoing myocardial ischemia reperfusion (IR). All values are expressed as means ± S.D., *n* = 8. ^∗^*P* < 0.05 versus the other groups.

After myocardial IR, the BAL fluid was collected and analyzed by various commercial cytokine kits. As shown in Figure 5, the levels of leukocyte count (a), TNF-*α* (b), IL-6 (c), and IL-8 (d) in BAL fluid in diabetic rats were significantly higher than that in control rats (all *P* < 0.05), suggesting the presence of severe inflammatory reaction in diabetic rats after myocardial IR insult. The autophagy inducer Rap significantly decreased leukocyte count and these inflammatory cytokine levels, but the autophagy inhibitor 3-MA have exactly the reverse effects.

We next investigated the influence of autophagy on pulmonary oxidative stress in diabetic rats after myocardial IR. As shown in Figures 5(e) and 5(f), diabetic rats subjected to myocardial IR showed significantly decreased SOD activity and increased 15-F2t-IsoP formations as compared with that in control rats. All these changes were attenuated by Rap but were further increased by 3-MA.

### 3.5. Effects of Rap and 3-MA on LC-3II/LC-3I and Beclin-1 Expression in Diabetic Rats Subjected to Myocardial IR

**Figure 6:**
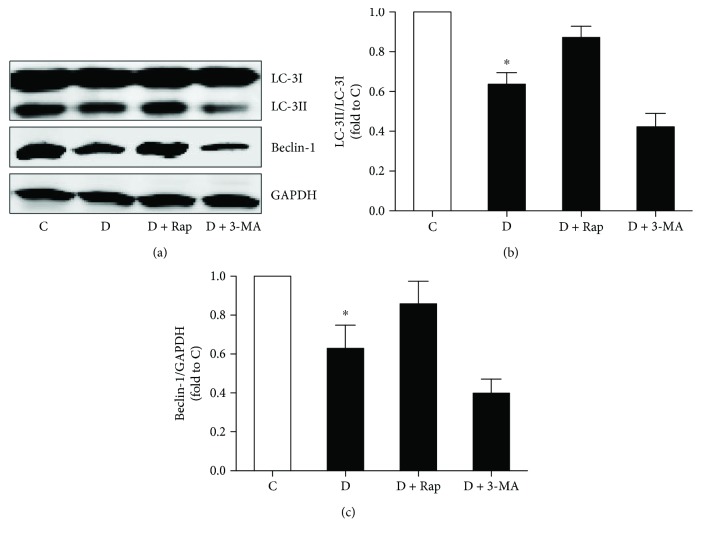
Effects of rapamycin (Rap) and 3-methyladenine (3-MA) on the pulmonary expression of LC-3II and Beclin-1 in diabetic rats. Control (C) and diabetic rats (D) pretreated with intraperitoneal 3-MA (15 mg/kg) 30 min or intravenous Rap (0.25 mg/kg) 15 min before ischemia or equal volume of saline were subjected to 30 min of myocardial ischemia followed by 2 h of reperfusion. (a) Representative Western blot demonstrating LC-3II, LC-3I, and Beclin-1 expression with GAPDH as loading control. (b) LC-3II/LC-3I ratio was calculated by relative densitometric values and expressed as fold to C group. (c) Beclin-1 express was calculated by relative densitometric values and expressed as fold to C group. All values are expressed as mean means ± S.D., *n* = 8. ^∗^*P* < 0.05 versus the other groups.

We then detected autophagy-related protein LC-3II/LC-3I and Beclin-1 in lung tissues after myocardial IR. As shown in Figures 6(a) and 6(b), the ratio of LC-3II/LC-3I and Beclin-1 expression in diabetic rats subjected to myocardial IR were lower than that in control rats, which were significantly increased by Rap but were further decreased by 3-MA treatment.

## 4. Discussions

In the present study, we have demonstrated that diabetic lungs are more vulnerable to myocardial IR. We provide evidence that ALI secondary to myocardial IR is associated with impaired autophagy status. Improving autophagy by Rap attenuates ALI secondary to myocardial IR in STZ-administrated diabetic rats, possibly through inhibiting inflammatory cytokine releases and reducing oxidative stress, but inhibition of autophagy with 3-MA has adverse effects, indicating that autophagy plays a critical role in attenuating ALI secondary to myocardial IR. To our knowledge, this is the first study to investigate the effects of autophagy on inflammatory reaction and oxidative stress in diabetic lungs subjected to myocardial IR.

Diabetes remains a substantial global health threat due to the systemic damages of hyperglycemia, which may lead to the development and progression of many representative complications, such as diabetic cardiomyopathy, diabetic nephropathy, diabetic retinopathy, diabetic foot, and diabetic neuropathy [1, 16]. Additionally, diabetes may noticeably accelerate the deterioration of respiratory function due to the destruction of normal anatomy and biology in diabetic lung [2]. Diabetes disturbs ventilatory function through decreasing respiratory muscle strength [17]. Besides, the abnormal glycation causes the basilar membrane thickening and the vascular smooth muscle cell hyperplasia, which undermine the ratio of ventilation and blood flow. Chronic hyperglycemia also induces dysbacteria in the lungs, which increases the chance of pulmonary infections and consequently leads to inflammatory infiltration [18]. The consequence of these pulmonary involvements confers fragility to diabetic lungs, when they underwent an acute or chronic pulmonary and/or cardiac disease (e.g., myocardial IR). Consistent with the previous studies [7], our present study have demonstrated that diabetic lungs are more vulnerable to myocardial IR, indicated by our results that diabetic rats subjected to myocardial IR showed serious ALI with higher lung injury score and WET/DRY ratio, and lower PaO_2_ as compared with that in control rats.

Patients with diabetes correlate with a much higher risk of cardiovascular disease and much worse prognosis of myocardial infarction [19, 20]. And, acute myocardial ischemia is considered as a major factor of acute severe pulmonary arterial hypertension [21] and pulmonary hemodynamics [22], resulting in ALI. There are many mechanisms that have been investigated in ALI, which have concocted different hypotheses to understand the mechanisms of pulmonary dysfunction induced by myocardial IR. In diabetic conditions, the lung tissue presented significant increase of reactive oxygen species (ROS) production coupled with a significant decrease of endogenous antioxidant, such as SOD activity, nicotinamide adenine dinucleotide phosphate (NADPH), and glutathione (GSH) [23]. Further, there are more proinflammatory cytokines in circulation of diabetic mice, as well as increased neutrophil and macrophage infiltration, proinflammatory cytokine production in lung tissue, which aggregates lung dysfunction and ALI [24, 25]. Reducing pulmonary inflammatory responses and oxidative stress could ultimately relieve lung injury in diabetic rats [26]. Experimental studies have also demonstrated that myocardial IR could cause lung complications involved in oxidative stress and inflammation [3, 27, 28]. Hence, redox homeostasis and inflammation have been implicated in diabetic lungs and thus diabetic lungs are more vulnerable to myocardial IR. In the present study, we suggested redundant lipid peroxidation products and proinflammation cytokines, accompanied with more antioxidative and anti-inflammation species depletion in lung tissue from diabetic rats subjected to myocardial IR, which resulted in a rapid loss of pulmonary function and severe lung injury.

Autophagy occurs at basal level, which occupies a pivotal position in preserving intracellular homeostasis and surviving via degrading and recycling intracellular proteins and damaged organelles. Although many investigations revealed conflicting results as to whether autophagy protects myocardium from IR [29], autophagy have been demonstrated to participate in lung IR injury [9], showing that autophagy was elevated during the ischemia period and enhanced significantly during reperfusion. Besides, the levels of autophagy induced by cold ischemia preservation for lung transplantation was initiated at 3 h, peaked at 6 h, and declined thereafter [30]. Accordingly, it is reasonable that the severity and duration of IR could elicit a controllable or uncontrollable autophagy, which drives cells to survival or demise. In the present study, we found pulmonary autophagy status was impaired in both control and diabetic rats subjected to 30 min ischemia followed by 2 h of reperfusion, this impaired autophagy status was also found in lung tissues subjected to pulmonary IR insult [11]. We speculated these conflict results may be involved in the long duration of myocardial IR resulting in declined pulmonary autophagy and might stem from the diversity in the experimental protocol.

There is a prevalent view that the reduction of autophagy in type 1 diabetic myocardium may be an adaptive response to prevent excessive autophagic degradation of cellular components [31, 32], but little studies outline the autophagic alteration in diabetic lung with or without the exposure of myocardial IR. In the present study, pulmonary autophagy was impaired in STZ-induced diabetic rats, which was further reduced by myocardial IR, as evidenced by further decreased expression of Beclin-1 and LC3II/LC3I ratio. Increasing evidences have indicated the involvement of autophagy, oxidative stress, and inflammation in lung injury [33], but the relationship among them is not very cleared, especially in ALI secondary to myocardial IR in diabetes. In agreement with previous studies, our present study showed improving autophagy status could exert beneficial effects to attenuate lung injury through markedly alleviating oxidative stress and inflammation [34–36], but the autophagy inhibitor had adverse effects because of further downregulation of autophagy, which resulted in more severe oxidative stress and systemic inflammation. Therefore, restoring autophagy status may be an effective means to attenuate ALI induced by myocardial IR in diabetes through inhibiting oxidative stress and inflammation.

In summary, our present study demonstrated that diabetes-impaired autophagy status is associated with severe ALI secondary to myocardial IR in diabetic rats. Improving autophagy status attenuates ALI induced by myocardial IR possibly through inhibiting inflammation and oxidative stress. Maintaining autophagy homeostasis may therefore represent a novel therapeutic means to attenuate lung injury in diabetes.

## Figures and Tables

**Table 1:** General characteristics in control and diabetic rats.

	Control	Diabetes
Body weight (g)	485.0 ± 24.6	279.3 ± 29.5^∗^
Food consumption (g/kg/day)	74.5 ± 14.3	186.8 ± 28.5^∗^
Water intake (mL/kg/day)	135.5 ± 17.3	878.5 ± 68.0^∗^
Blood glucose (mM)	6.4 ± 0.2	26.2 ± 5.9^∗^

All values are expressed as means ± S.D., *n* = 8; water intake and food consumption values were the average value during this study. Blood glucose and body weight were measured before sacrifice. ^∗^*P* < 0.05 versus control group.
